# Cabells Scholarly Analytics

**DOI:** 10.5195/jmla.2018.403

**Published:** 2018-04-01

**Authors:** Lilian Hoffecker

**Affiliations:** Health Sciences Library, University of Colorado Anschutz Medical Campus, Aurora, CO

## INTRODUCTION

Librarians are frequently asked for help in finding an appropriate journal for a working manuscript. Tools exist to match a manuscript title or abstract with a journal in a similar subject area, but a journal’s suitability depends on more than a fit in field of study. Typical questions authors ask include: How frequently is an issue published? Who are the editors and published authors? How influential is the publication? How competitive is it? Can I trust the journal and its publisher?

Cabells Scholarly Analytics is a database of journals describing peer-review policy, fees, quality metrics, and many more features that researchers find helpful in making decisions about where to publish. Consisting of the Whitelist of reputable journals and the Blacklist of questionable journals, Cabells aims to become a reliable source of information on the quality, competiveness, visibility, and integrity of journals. The Blacklist, specifically, is a dispassionate, potentially one-stop resource to help authors identify problematic journals. There is room for improvement, however, especially for the Whitelist, in accurately categorizing journals by discipline and transparently showing the methodology of calculated indices.

## OVERVIEW

Cabells has offered publishing directories for researchers since the 1970s, focusing on fields in business but expanding over the decades to other disciplines. The database comprises two lists: the Whitelist of reputable journals and its most recent product, the Blacklist of potentially questionable journals. The Whitelist consists of 11,000 journals spanning 18 disciplines, mostly in the social sciences (including library science) and the physical sciences. The health sciences are not as thoroughly represented, with the exception of nursing, health administration, and some behavioral health specialties. The Blacklist contains more than 6,800 journals as of late fall 2017 and covers all disciplines, including the health sciences. The intended audience for the Cabells database includes academics, librarians, administrators, and educators.

The Whitelist provides descriptive information for each journal, guiding authors to those journals that correspond to their publication needs, while the Blacklist directs authors away from journals with problematic practices. In a separate service, which this review does not cover, Cabells works with Editage to help authors write and edit their manuscripts [1].

The Whitelist and the Blacklist are navigated separately. The user can search the Whitelist for particular journals and filter by discipline or topic, publisher, International Standard Serial Number (ISSN), open access, and various metrics. The Blacklist search engine is more limited, allowing users to search by keyword, publisher, open access, and ISSN. Users cannot, however, sort the Whitelist by discipline, for example, or the Blacklist by number of violations.

## FEATURES OF THE WHITELIST AND THE BLACKLIST

Journals make it onto the Whitelist by invitation only [2]. Cabells considers not only criteria such as audience and society sponsorship, but also criteria related to quality (such as rigor in peer review) and integrity (such as clear statements about fees). Any journal on the Whitelist has, therefore, passed a series of checks on quality assessment, transparency of policies, and ethics.

A journal profile in the Whitelist includes its disciplinary focus, frequency of publication, editor contacts, and launch date, but it also reports journal features that are not easily located or even available on publisher websites. These include the percentage of invited articles, peer-review policy and review time, number and type of reviewers (internal versus external), and plagiarism screening. Every journal is evaluated by at least three trained reviewers with appropriate educational credentials in business, psychology, engineering, medicine, computer science, and other disciplines.

In addition to descriptive information, Cabells presents metrics related to quality and visibility, namely the impact factor from Journal Citation Reports, the Altmetric score, and its own Cabells classification index (CCI). Like the impact factor, the CCI is citation-based but uses Scopus as its data source, where available, and like the other metrics, is an approximation of influence and quality in a subject area [3]. A journal can have multiple CCIs if it encompasses multiple disciplines and multiple topics in the disciplines. The CCI is calculated using the average citation rate across three years and *z*-transformed (standardized) in a discipline or topic [4]. For example, the *Journal of the Medical Library Association (JMLA)* is classified under two disciplines, Educational Technology & Library Science (ETLS) and Health Administration. For ETLS, the *JMLA* is further classified under the topic of “medical libraries.” The CCI for the *JMLA* in the broader discipline of ETLS is 69%, while specifically in “medical libraries,” it is 72%. The CCI is a percentile, so 69% of publications in the ETLS discipline fall below the *JMLA* in quality, while 72% of publications in the topic of “medical libraries” fall below the *JMLA*.

Another unique Whitelist metric is the “Difficulty of Acceptance” percentile. Like the CCI, it depends on discipline and is based on the average number of times an author from a “high performing institution” publishes in a journal in a discipline. A percentile less than or equal to 10% is regarded as rigorous, 11%–20% is very difficult, and anything greater than 20% is simply difficult.

While authors may feel assured by the legitimacy of journals on the Whitelist, they are advised to stay away from those on the Blacklist. The list is based on sixty-five criteria called “Behavioral Indicators,” which are used to evaluate individual journals, not publishers [5]. The criteria fit in eight categories: Integrity, Peer Review, Website, Publication Practices, Indexing & Metrics, Fees, Access & Copyright, and Business Practices. Some criteria are easy to verify (e.g., “the journal uses a fake ISSN”), but others require research (e.g., “insufficient resources are spent on preventing and eliminating author misconduct”). Reviewers examine every journal and report scores by the number of “violations.” Blacklisted journals and their publishers are given the opportunity to appeal.

## DISCUSSION

The CCI is a key part of the Whitelist, for it offers a quick way for authors to assess a journal, but interpretation of the index is unclear: What does 69% or 72% CCI for the *JMLA* actually mean? Is the percentile based purely on citation counts, or is there a method for weighting by the type of article that cites a *JMLA* article (e.g., news items versus research articles)? What about self-citations? There are journals with a CCI of 100% (e.g., *Alzheimer’s & Dementia*), which is technically impossible since percentiles include the item being scored. Most likely the percentile scores are rounded, but the description of the calculation method is not detailed enough to answer this and other questions.

Furthermore, the disciplinary categories for the journals, which have an important role in the quality assessments, need to be reevaluated. For example, the *JMLA* is categorized appropriately under the ETLS discipline, but also, surprisingly, under Health Administration. For the latter, the CCI is 43%, an index considerably lower than the 69% for ETLS. Scopus is the source of subject categories, though journal editors may request specific disciplines and topics [6].

In addition to affecting the CCI, imprecise categorization can be misleading to an author who may rely on the designated disciplines and topics to submit an article in a particular field. For example, *Medical Teacher,* an education journal for the health professions, is listed under the broad category of ETLS and the unexpected topics of “medical libraries” and “academic libraries” within ETLS [7]. The CCIs are high at 88% and 97%, respectively, for topics that are not major subjects of the journal.

Similar to the CCI, the calculation methodology for the Difficulty of Acceptance metric is not clearly described: Which institutions are considered “high performing institutions?” How is “high performing” defined? How does Cabells select the authors from these institutions? Does a “rigorous” journal have a high CCI?

The profiles for each Blacklist journal shows the number and type of violations, but Cabells reports that quantity is not the sole consideration, and in fact, criteria are weighted [8]. Deceptive practices (e.g., an article appearing in more than one journal) are weighted more heavily than mere carelessness (e.g., poor copyediting of the website), but there is no indication of such differentiation in the database [9]. New journals just starting out might fail criteria such as “no policies for digital preservation” or the website has “dead links,” and for those in countries where English is not the primary language, the journal description might include “poor grammar and/or spelling.” The ability to sort or filter by number and type of violation (e.g., all journals that have faked their ISSN) would be a useful enhancement for the Blacklist.

## CONCLUSIONS

A primary concern of the Whitelist is inaccurate organization of journals into subject categories. Appropriate categorization is essential, because it affects the metrics and how they are interpreted. Related to this, transparency associated with how these metrics are calculated is necessary, because users have to trust (a central theme of this database) the mark of quality or legitimacy. Including the impact factor to further support the data-driven nature of the Whitelist can be helpful but only if the *ranking* of a journal by impact factor in its discipline from Journal Citation Reports is also included. The value alone says very little without comparison to other related journals. Fortunately, the Altmetric score, once clicked, is enhanced with information about the types of media (social media, national media, blogs, etc.) that contribute to the score.

Another matter of potential concern, given the volume of journals and thoroughness of the reviews, is completeness and currency. The Whitelist selection policy reassures users that audits are performed annually and when journals change their editorial practices [2, 6]. The Blacklist launched in May 2017 with 3,900 journals, but by the time of this review, the list grew to 6,800, indicating that Cabells evaluated and added as many as 2,900 journals within a few months [10]. The question remains whether updates in both lists can be kept up at this pace. When consulted by faculty, can librarians have confidence in the currency of the Blacklist or the continuing quality and influence implied by a Whitelist journal’s CCI?

Despite reservations, the Blacklist in particular is a much-needed objective resource. Over time, with refinement and openness about its methodology, both lists of Cabells Scholarly Analytics can become invaluable to authors.
